# 4-Benzyl-1-(4-nitro­phen­yl)-1*H*-1,2,3-triazole: crystal structure and Hirshfeld analysis

**DOI:** 10.1107/S2056989017014748

**Published:** 2017-10-20

**Authors:** Julio Zukerman-Schpector, Sofia Dallasta Pedroso, Lucas Sousa Madureira, Márcio Weber Paixão, Akbar Ali, Edward R. T. Tiekink

**Affiliations:** a Laboratório de Cristalografia, Esterodinâmica e, Modelagem Molecular, Departamento de Química, Universidade Federal de São Carlos, 13565-905 São Carlos, SP, Brazil; b Departamento de Química, Universidade Federal de São Carlos, 13565-905 São Carlos, SP, Brazil; c Research Centre for Crystalline Materials, School of Science and Technology, Sunway University, 47500 Bandar Sunway, Selangor Darul Ehsan, Malaysia

**Keywords:** crystal structure, 1,2,3-triazole, Hirshfeld surface analysis

## Abstract

The mol­ecule in the title compound, C_15_H_12_N_4_O_2_, has a twisted *L*-shape with the dihedral angle between the aromatic rings of the N-bound benzene and C-bound benzyl groups being 70.60 (9)°. The nitro group is co-planar with the benzene ring to which it is connected [C—C—N—O torsion angle = 0.4 (3)°]. The three-dimensional packing is stabilized by a combination of methyl­ene-C—H⋯O(nitro), methyl­ene-C—H⋯π(phen­yl), phenyl-C—H⋯π(triazol­yl) and nitro-O⋯π(nitro­benzene) inter­actions, along with weak π(triazol­yl)–π(nitrobenzene) contacts [inter-centroid distance = 3.8386 (10) Å]. The importance of the specified inter­molecular contacts has been verified by an analysis of the calculated Hirshfeld surface.

## Abstract 2

In the title compound, the 1,2,3-triazoyl ring is flanked by nitro­benzene and benzyl substituents with the dihedral angle of 70.60 (9)° between rings indicating a twisted l-shape for the mol­ecular conformation.

## Chemical context   

The 1,2,3-triazoles comprise an important class of mol­ecules, having a number of applications in biology and materials science. As reviewed recently, 1,2,3-triazoles display various potential pharmaceutical properties including anti-cancer, anti-viral, anti-tuberculosis and anti-microbial activities (Tron *et al.*, 2008 ▸; Thirumurugan *et al.*, 2013 ▸). The 1,2,3-triazole chromo­phore can function as a most useful scaffold in bio-conjugation owing to its rigid framework, stability, and, crucially, water-solubility (Jewett & Bertozzi, 2010 ▸; Holub & Kirshenbaum, 2010 ▸). Further applications are known in the fields of dyes, photostabilizers and as agrochemicals (Golas & Matyjaszewski, 2010 ▸; Qin *et al.*, 2010 ▸). Very recently, a new and efficient synthesis for a metal-free and regioselective synthesis of 1,4-disubstituted 1,2,3-triazoles was described (Ali *et al.*, 2014 ▸). Among the compounds synthesized in that study was the title compound, (I). Herein, the crystal and mol­ecular structures of (I) are described along with an analysis of the Hirshfeld surface.
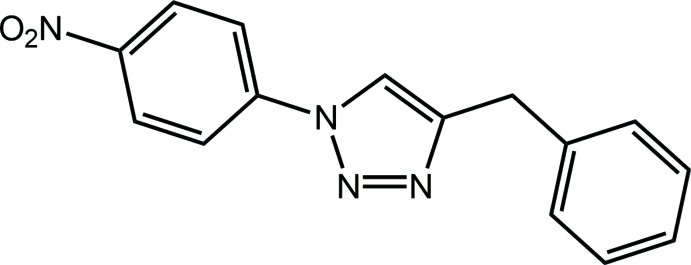


## Structural commentary   

**Figure 1:**
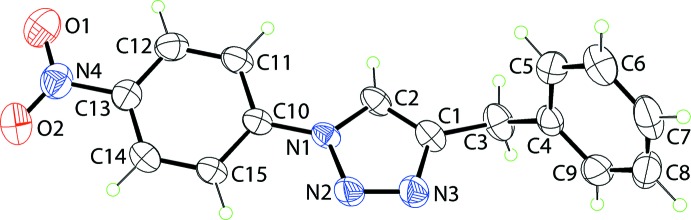
The mol­ecular structure of (I), showing the atom-labelling scheme and displacement ellipsoids at the 35% probability level.

The mol­ecular structure of (I), Fig. 1 ▸, comprises a central, strictly planar 1,2,3-triazolyl ring (r.m.s. deviation of the five fitted atoms = 0.001 Å) flanked by C- and N-bound benzyl and 4-nitro­benzene substituents, respectively. The dihedral angle between the five-membered ring and phenyl ring is 83.23 (10)°, indicating a near perpendicular relationship. By contrast, the benzene ring is closer to co-planar to the triazolyl ring, forming a dihedral angle of 13.95 (9)°. The dihedral angle between the outer rings is 70.60 (9)°, indicating that the mol­ecule has a skewed-shape based on the letter *L*. The nitro group is co-planar with the benzene ring to which it is bound as seen in the value of the C12—C13—N4—O1 torsion angle of 0.4 (3)°.

## Supra­molecular features   

**Figure 2:**
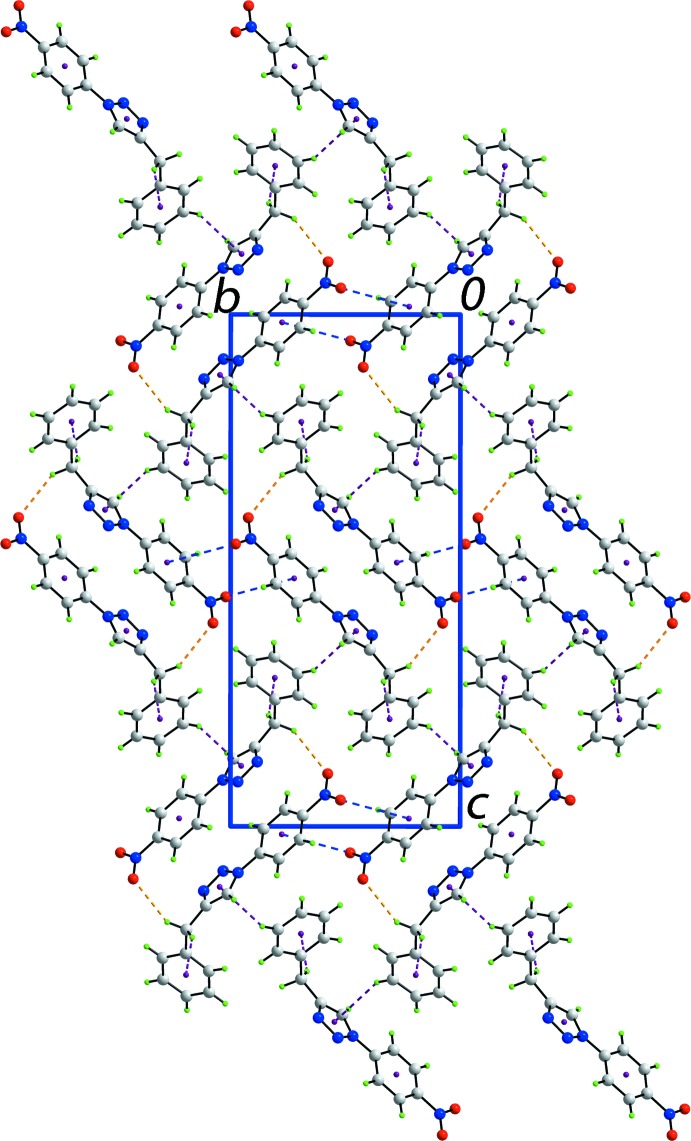
A view of the unit-cell contents in projection down the *a* axis. The C—H⋯O, C—H⋯π and nitro-O⋯π contacts are shown as orange, purple and blue dashed lines, respectively.

The mol­ecular packing of (I) features methyl­ene-C—H⋯O(nitro), methyl­ene-C—H⋯π(phen­yl), phenyl-C—H⋯π(triazol­yl) and nitro-O⋯π(nitro­benzene) inter­actions, Table 1 ▸; the latter inter­actions have been described as being important in stabilizing the crystal packing of nitro-containing compounds (Huang *et al.*, 2008 ▸). The C—H⋯O and nitro-O⋯π inter­actions occur between centrosymmetrically related mol­ecules while the C—H⋯π(phen­yl) contacts occur along the *a*-axis direction and the C—H⋯π(triazol­yl) contacts along the *b*-axis direction and, all taken together, consolidate the three-dimensional architecture, Fig. 2 ▸. Within the specified framework, weak π(triazol­yl)–π(nitro­benzene)^i^ inter­actions occur with the inter-centroid distance = 3.8386 (10) Å, inter-planar angle = 13.95 (9)° for symmetry code: (i) 1 − *x*, − y, 2 − *z*.

## Hirshfeld surface analysis   

**Figure 3:**
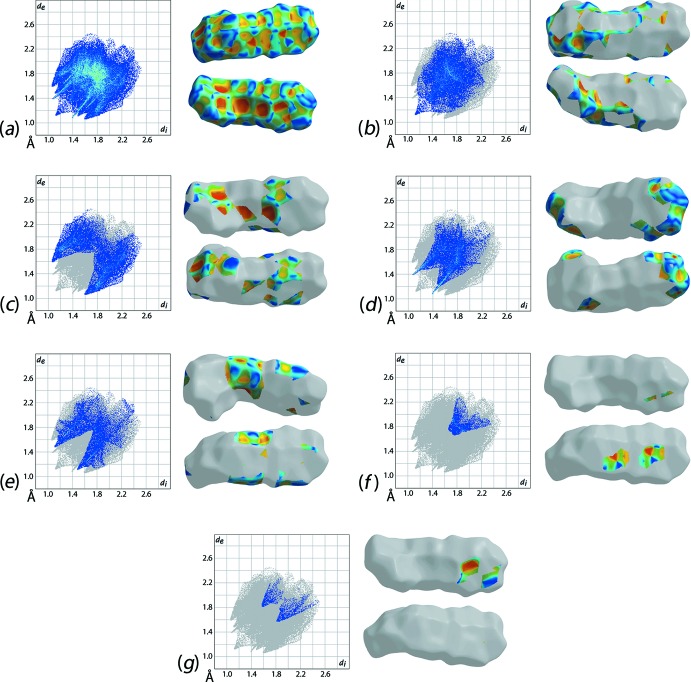
(*a*) The full two-dimensional fingerprint plot for (I) and two views of the Hirshfeld surface mapped over the shape-index property, and fingerprint plots delineated into (*b*) H⋯H, (*c*) C⋯H/H⋯C, (*d*) O⋯H/H⋯O, (*e*) N⋯H/H⋯N, (*f*) C⋯N/N⋯C and (*g*) C⋯O/O⋯C inter­atomic contacts along with two views of Hirshfeld surface mapped over shape-index.

The study of the Hirshfeld surface and inter­molecular inter­actions of (I) has been carried out using standard parameters of the *CrystalExplorer* package (Wolff *et al.*, 2012 ▸) and using similar protocols as in earlier studies (Zukerman-Schpector *et al.*, 2017 ▸). In (I), the Hirshfeld surface is controlled by attractive inter­actions such as non-conventional C—H⋯π, C—H⋯O, C—H⋯N hydrogen bonds and π–π inter­actions. The aforementioned contacts contribute around 70% to the overall surface area, Fig. 3 ▸ and Table 2 ▸. The repulsive H⋯H inter­actions account for the remaining 30%, Fig. 3 ▸ *b*. These observations may be rationalized in terms of the structure having electron-rich groups, *i.e*. the three aromatic rings and the nitro substituent, for which the electron densities are highly delocal­ized allowing them to have significant overlap in the mol­ecular packing.

As attractive inter­actions, the C⋯H/H⋯C contacts contribute a significant role (26.1%) to the overall surface area. These contacts arise mainly from C—H⋯π contacts spread over the entire mol­ecule in which all rings, *i.e.* the triazole, nitro­benzene and benzyl rings, function as H-atom acceptors, Tables 1 ▸ and 3 ▸, and Fig. 3 ▸ *c*. The O⋯H/H⋯O contacts contribute 21.0% to the Hirshfeld surface area. In essence, this arises owing to non-conventional C—H⋯O hydrogen bonds, Fig. 3 ▸ *d*. There are two different H-donor carbon atoms participating in the weak C—H⋯O inter­actions, one of which is the methyl­ene-C3 atom, Table 1 ▸, and the other being the nitro­benzene-C12 atom, Table 3 ▸. The N⋯H/H⋯N contacts contribute approximately 16% to the overall surface area, Fig. 3 ▸ *e*. Non conventional C—H⋯N hydrogen bonds are formed with nitro­benzene-C atoms as H-atom donors, Table 3 ▸ and Fig. 3 ▸ *f*. The C⋯N/N⋯C and C⋯O/O⋯C contacts contribute around 6% to the Hirshfeld surface, Table 2 ▸ and Fig. 3 ▸ *f* and *g*. Other surface contacts do not contribute significantly to the mol­ecular packing.

## Database survey   

There are only relatively few 1,2,3-triazole structures in the literature having N-bound aryl groups and C-bound alkyl substituents. The two mol­ecules closest to (I) have N-bound 4-chloro­benzene and C-bound *n*-butyl groups, *i.e*. (II) (Sarode *et al.*, 2016 ▸), and N-bound 4-nitro­benzene and *C*-bound *n*-hexyl groups, *i.e*. (III) (Muhammad *et al.*, 2015 ▸). In (II), the dihedral angle between the two planes is 22.59 (7)° and the *n*-butyl group is co-planar with the the five-membered ring as seen in the *sp* ^2^-C—C_quaternary_—C—C_methyl­ene_ = 0.06 (4)° and C_methyl­ene_—C—C—C_meth­yl_ = −177.39 (19)° torsion angles. In (III), the aromatic rings are considerably more co-planar, *cf*. (I) and (II), with the dihedral angle between them being 2.65 (8)°. With respect to the *n*-hexyl substituent, the structure of (III) resembles that of (I) in that the *sp* ^2^-C—C_quaternary_—C—C_methyl­ene_ torsion angle is −118.4 (3)°.

## Synthesis and crystallization   

The title compound was prepared as described in the literature (Ali *et al.*, 2014 ▸). Crystals of (I) for the X-ray study were obtained by slow evaporation from an ethyl acetate/*n*-hexane solution (5:1 *v*/*v*). ^1^H NMR (400 MHz, CDCl_3_) δ 7.70–7.65 (*m*, 2H), 7.59 (*s*, 1H), 7.51–7.45 (*m*, 2H), 7.42–7.32 (*m*, 3H), 7.31–7.21 (*m*, 2H), 4.17 (*s*, 2H). ^13^C NMR (100 MHz, CDCl_3_) δ = 148.5, 138.8, 137.2, 129.6, 128.8, 128.7, 128.5, 126.6, 120.42, 119.6, 32.3 ppm. ESI–MS (*m*/*z*) calculated for C_15_H_12_N_4_O_2_ [*M* + H]^+^ 281.1038, found 281.1039.

## Refinement details   

Crystal data, data collection and structure refinement details are summarized in Table 4 ▸. The carbon-bound H atoms were placed in calculated positions (C—H = 0.93–0.97 Å) and were included in the refinement in the riding-model approximation, with *U*_iso_(H) set to 1.2*U*_eq_(C).

## Supplementary Material

Crystal structure: contains datablock(s) I, global. DOI: 10.1107/S2056989017014748/hg5498sup1.cif

Structure factors: contains datablock(s) I. DOI: 10.1107/S2056989017014748/hg5498Isup2.hkl

Supporting information file. DOI: 10.1107/S2056989017014748/hg5498Isup3.cml

CCDC reference: 1579464

Additional supporting information:  crystallographic information; 3D view; checkCIF report

## Figures and Tables

**Table 1:** Hydrogen-bond geometry (Å, °) *Cg*1–*Cg*3 are the centroids of the N1–N3,C1,C2, C4–C9 and C10-C15 rings, respectively.

*D*—H⋯*A*	*D*—H	H⋯*A*	*D*⋯*A*	*D*—H⋯*A*
C3—H3*B*⋯O2^i^	0.97	2.58	3.452 (3)	150
C3—H3*A*⋯*Cg*2^ii^	0.97	2.96	3.857 (2)	154
C8—H8⋯*Cg*1^iii^	0.93	2.86	3.665 (3)	146
N4—O1⋯*Cg*3^iv^	1.21 (1)	3.67 (1)	4.1254 (19)	103 (1)

Symmetry codes: (i) 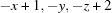; (ii) 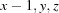; (iii) 
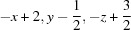
; (iv) 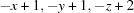.

**Table 2:** Percentage contributions of inter­atomic contacts to the Hirshfeld surface for (I)

Contact	Percentage contribution
H⋯H	28.7
C⋯H/H⋯C	26.1
O⋯H/H⋯O	21.0
N⋯H/H⋯N	15.6
C⋯N/N⋯C	3.9
C⋯O/O⋯C	2.4
others	2.3

**Table 3:** Summary of short inter­atomic contacts (Å) in (I)

Contact	Distance	Symmetry operation
H15⋯H15	2.54	2 − *x*, −*y*, 2 − *z*
C7⋯H11	2.78	1 − *x*, − 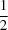 + *y*, 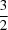 − *z*
O1⋯H12	2.62	−*x*, 1 − *y*, 2 − *z*
O1⋯C13	3.386 (3)	1 − *x*, 1 − *y*, 2 − *z*
C15⋯N1	3.413 (2)	1 − *x*, −*y*, 2 − *z*
H15⋯N2	2.71	2 − *x*, −*y*, 2 − *z*
H14⋯N3	3.00	2 − *x*, −*y*, 2 − *z*

**Table 4:** Experimental details

Crystal data	
Chemical formula	C_15_H_12_N_4_O_2_
*M*_r_	280.29
Crystal system, space group	Monoclinic, *P*2_1_/*c*
Temperature (K)	293
*a*, *b*, *c* (Å)	5.1962 (1), 10.7814 (3), 24.0067 (6)
β (°)	90.256 (2)
*V* (Å^3^)	1344.90 (6)
*Z*	4
Radiation type	Mo *K*α
μ (mm^−1^)	0.10
Crystal size (mm)	0.46 × 0.26 × 0.14

Data collection	
Diffractometer	Bruker APEXII CCD
Absorption correction	Multi-scan (*SADABS*; Sheldrick, 1996 ▸)
*T*_min_, *T*_max_	0.695, 0.745
No. of measured, independent and observed [*I* > 2σ(*I*)] reflections	9104, 2450, 1881
*R*_int_	0.023
(sin θ/λ)_max_ (Å^−1^)	0.603

Refinement	
*R*[*F* ^2^ > 2σ(*F* ^2^)], *wR*(*F* ^2^), *S*	0.040, 0.106, 1.06
No. of reflections	2450
No. of parameters	190
H-atom treatment	H-atom parameters constrained
Δρ_max_, Δρ_min_ (e Å^−3^)	0.16, −0.18

Computer programs: *APEX2* and *SAINT* (Bruker, 2009 ▸), *SIR2014* (Burla *et al.*, 2015 ▸), *SHELXL2014* (Sheldrick, 2015 ▸), *ORTEP-3 for Windows* (Farrugia, 2012 ▸), *DIAMOND* (Brandenburg, 2006 ▸), *MarvinSketch* (ChemAxon, 2010 ▸) and *publCIF* (Westrip, 2010 ▸).

## supplementary crystallographic information

### Crystal data

**Table:**

C_15_H_12_N_4_O_2_	*D*_x_ = 1.384 Mg m^−^^3^
*M**_r_* = 280.29	Melting point = 371–373 K
Monoclinic, *P*2_1_/*c*	Mo *K*α radiation, λ = 0.71073 Å
*a* = 5.1962 (1) Å	Cell parameters from 2937 reflections
*b* = 10.7814 (3) Å	θ = 3.2–25.0°
*c* = 24.0067 (6) Å	µ = 0.10 mm^−^^1^
β = 90.256 (2)°	*T* = 293 K
*V* = 1344.90 (6) Å^3^	Irregular, yellow
*Z* = 4	0.46 × 0.26 × 0.14 mm
*F*(000) = 584	

### Data collection

**Table:**

Bruker APEXII CCD diffractometer	1881 reflections with *I* > 2σ(*I*)
φ and ω scans	*R*_int_ = 0.023
Absorption correction: multi-scan (SADABS; Sheldrick, 1996)	θ_max_ = 25.4°, θ_min_ = 1.7°
*T*_min_ = 0.695, *T*_max_ = 0.745	*h* = −6→6
9104 measured reflections	*k* = −12→12
2450 independent reflections	*l* = −28→28

### Refinement

**Table:**

Refinement on *F*^2^	0 restraints
Least-squares matrix: full	Hydrogen site location: inferred from neighbouring sites
*R*[*F*^2^ > 2σ(*F*^2^)] = 0.040	H-atom parameters constrained
*wR*(*F*^2^) = 0.106	*w* = 1/[σ^2^(*F*_o_^2^) + (0.0383*P*)^2^ + 0.3766*P*] where *P* = (*F*_o_^2^ + 2*F*_c_^2^)/3
*S* = 1.06	(Δ/σ)_max_ = 0.001
2450 reflections	Δρ_max_ = 0.16 e Å^−^^3^
190 parameters	Δρ_min_ = −0.18 e Å^−^^3^

### Special details

**Table:**

Geometry. All esds (except the esd in the dihedral angle between two l.s. planes) are estimated using the full covariance matrix. The cell esds are taken into account individually in the estimation of esds in distances, angles and torsion angles; correlations between esds in cell parameters are only used when they are defined by crystal symmetry. An approximate (isotropic) treatment of cell esds is used for estimating esds involving l.s. planes.

### Fractional atomic coordinates and isotropic or equivalent isotropic displacement parameters (Å^2^)

**Table:**

	*x*	*y*	*z*	*U*_iso_*/*U*_eq_	
C1	0.6006 (3)	−0.10629 (18)	0.84523 (7)	0.0532 (4)	
C2	0.4648 (3)	−0.01241 (18)	0.86767 (6)	0.0531 (4)	
H2	0.3030	0.0154	0.8566	0.064*	
C3	0.5294 (4)	−0.1916 (2)	0.79835 (7)	0.0672 (5)	
H3A	0.3644	−0.1662	0.7832	0.081*	
H3B	0.5100	−0.2750	0.8129	0.081*	
C4	0.7242 (3)	−0.19387 (16)	0.75201 (7)	0.0519 (4)	
C5	0.7534 (4)	−0.09304 (18)	0.71743 (8)	0.0646 (5)	
H5	0.6472	−0.0244	0.7220	0.078*	
C6	0.9359 (4)	−0.0918 (2)	0.67636 (8)	0.0762 (6)	
H6	0.9520	−0.0224	0.6536	0.091*	
C7	1.0933 (4)	−0.1905 (3)	0.66847 (9)	0.0803 (7)	
H7	1.2195	−0.1884	0.6411	0.096*	
C8	1.0641 (4)	−0.2926 (3)	0.70110 (10)	0.0836 (7)	
H8	1.1682	−0.3616	0.6954	0.100*	
C9	0.8791 (4)	−0.29477 (19)	0.74304 (8)	0.0695 (5)	
H9	0.8607	−0.3651	0.7651	0.083*	
C10	0.5585 (3)	0.13033 (15)	0.94757 (6)	0.0444 (4)	
C11	0.3575 (3)	0.21075 (18)	0.93671 (7)	0.0577 (5)	
H11	0.2615	0.2024	0.9041	0.069*	
C12	0.2992 (4)	0.30319 (18)	0.97405 (8)	0.0627 (5)	
H12	0.1625	0.3569	0.9674	0.075*	
C13	0.4468 (3)	0.31461 (16)	1.02137 (7)	0.0541 (4)	
C14	0.6508 (3)	0.23726 (17)	1.03240 (7)	0.0562 (4)	
H14	0.7494	0.2478	1.0645	0.067*	
C15	0.7071 (3)	0.14397 (17)	0.99526 (6)	0.0511 (4)	
H15	0.8439	0.0904	1.0021	0.061*	
N1	0.6118 (2)	0.03310 (13)	0.90952 (5)	0.0451 (3)	
N2	0.8341 (3)	−0.03164 (15)	0.91281 (6)	0.0583 (4)	
N3	0.8269 (3)	−0.11635 (15)	0.87374 (6)	0.0628 (4)	
N4	0.3812 (4)	0.41242 (16)	1.06168 (7)	0.0729 (5)	
O1	0.1984 (4)	0.47864 (19)	1.05159 (8)	0.1170 (7)	
O2	0.5137 (4)	0.42327 (16)	1.10290 (7)	0.1031 (6)	

### Atomic displacement parameters (Å^2^)

**Table:**

	*U*^11^	*U*^22^	*U*^33^	*U*^12^	*U*^13^	*U*^23^
C1	0.0419 (9)	0.0744 (12)	0.0432 (8)	−0.0121 (8)	0.0010 (7)	0.0016 (8)
C2	0.0367 (8)	0.0806 (13)	0.0420 (8)	−0.0047 (8)	−0.0035 (7)	0.0027 (8)
C3	0.0609 (11)	0.0851 (14)	0.0556 (10)	−0.0213 (10)	0.0005 (9)	−0.0091 (10)
C4	0.0513 (10)	0.0585 (11)	0.0458 (8)	−0.0051 (8)	−0.0084 (7)	−0.0099 (8)
C5	0.0728 (13)	0.0615 (12)	0.0596 (11)	0.0065 (10)	0.0030 (9)	−0.0019 (9)
C6	0.0879 (15)	0.0881 (16)	0.0526 (11)	−0.0147 (13)	0.0046 (10)	−0.0034 (10)
C7	0.0646 (13)	0.122 (2)	0.0539 (11)	−0.0053 (14)	0.0001 (10)	−0.0310 (13)
C8	0.0712 (14)	0.0994 (18)	0.0800 (14)	0.0278 (13)	−0.0165 (12)	−0.0410 (14)
C9	0.0803 (14)	0.0621 (12)	0.0660 (12)	0.0053 (11)	−0.0214 (10)	−0.0076 (10)
C10	0.0371 (8)	0.0573 (10)	0.0389 (8)	−0.0030 (7)	0.0038 (6)	0.0098 (7)
C11	0.0495 (10)	0.0753 (13)	0.0483 (9)	0.0074 (9)	−0.0065 (8)	0.0056 (9)
C12	0.0561 (11)	0.0688 (12)	0.0631 (11)	0.0122 (9)	0.0009 (9)	0.0083 (9)
C13	0.0581 (10)	0.0533 (10)	0.0509 (9)	−0.0034 (8)	0.0107 (8)	0.0048 (8)
C14	0.0568 (10)	0.0665 (11)	0.0454 (8)	−0.0074 (9)	−0.0031 (8)	0.0035 (8)
C15	0.0439 (9)	0.0627 (11)	0.0468 (9)	0.0007 (8)	−0.0050 (7)	0.0048 (8)
N1	0.0337 (7)	0.0630 (9)	0.0384 (6)	0.0005 (6)	−0.0008 (5)	0.0050 (6)
N2	0.0415 (8)	0.0786 (10)	0.0547 (8)	0.0099 (7)	−0.0091 (6)	−0.0088 (8)
N3	0.0518 (9)	0.0784 (11)	0.0582 (9)	0.0064 (8)	−0.0062 (7)	−0.0122 (8)
N4	0.0873 (13)	0.0647 (11)	0.0668 (11)	−0.0013 (10)	0.0104 (10)	−0.0005 (9)
O1	0.1292 (16)	0.1118 (14)	0.1100 (13)	0.0544 (13)	−0.0054 (12)	−0.0279 (11)
O2	0.1333 (15)	0.0942 (12)	0.0817 (11)	0.0065 (11)	−0.0145 (11)	−0.0274 (9)

### Geometric parameters (Å, º)

**Table:**

C1—C2	1.347 (2)	C9—H9	0.9300
C1—N3	1.362 (2)	C10—C11	1.382 (2)
C1—C3	1.499 (2)	C10—C15	1.386 (2)
C2—N1	1.3516 (19)	C10—N1	1.418 (2)
C2—H2	0.9300	C11—C12	1.375 (3)
C3—C4	1.507 (2)	C11—H11	0.9300
C3—H3A	0.9700	C12—C13	1.373 (2)
C3—H3B	0.9700	C12—H12	0.9300
C4—C9	1.371 (3)	C13—C14	1.374 (2)
C4—C5	1.377 (2)	C13—N4	1.472 (2)
C5—C6	1.371 (3)	C14—C15	1.376 (2)
C5—H5	0.9300	C14—H14	0.9300
C6—C7	1.356 (3)	C15—H15	0.9300
C6—H6	0.9300	N1—N2	1.3516 (18)
C7—C8	1.360 (3)	N2—N3	1.310 (2)
C7—H7	0.9300	N4—O2	1.209 (2)
C8—C9	1.395 (3)	N4—O1	1.212 (2)
C8—H8	0.9300		
			
C2—C1—N3	108.14 (15)	C4—C9—H9	119.8
C2—C1—C3	129.31 (16)	C8—C9—H9	119.8
N3—C1—C3	122.53 (17)	C11—C10—C15	120.48 (16)
C1—C2—N1	105.95 (14)	C11—C10—N1	119.46 (14)
C1—C2—H2	127.0	C15—C10—N1	120.06 (14)
N1—C2—H2	127.0	C12—C11—C10	120.02 (15)
C1—C3—C4	113.59 (14)	C12—C11—H11	120.0
C1—C3—H3A	108.8	C10—C11—H11	120.0
C4—C3—H3A	108.8	C13—C12—C11	118.71 (17)
C1—C3—H3B	108.8	C13—C12—H12	120.6
C4—C3—H3B	108.8	C11—C12—H12	120.6
H3A—C3—H3B	107.7	C12—C13—C14	122.19 (17)
C9—C4—C5	117.75 (18)	C12—C13—N4	118.55 (17)
C9—C4—C3	121.69 (18)	C14—C13—N4	119.26 (16)
C5—C4—C3	120.56 (17)	C13—C14—C15	119.00 (16)
C6—C5—C4	121.32 (19)	C13—C14—H14	120.5
C6—C5—H5	119.3	C15—C14—H14	120.5
C4—C5—H5	119.3	C14—C15—C10	119.57 (16)
C7—C6—C5	120.8 (2)	C14—C15—H15	120.2
C7—C6—H6	119.6	C10—C15—H15	120.2
C5—C6—H6	119.6	C2—N1—N2	109.62 (14)
C6—C7—C8	119.1 (2)	C2—N1—C10	129.48 (13)
C6—C7—H7	120.5	N2—N1—C10	120.88 (12)
C8—C7—H7	120.5	N3—N2—N1	107.26 (13)
C7—C8—C9	120.5 (2)	N2—N3—C1	109.03 (15)
C7—C8—H8	119.7	O2—N4—O1	123.3 (2)
C9—C8—H8	119.7	O2—N4—C13	118.35 (19)
C4—C9—C8	120.5 (2)	O1—N4—C13	118.30 (18)
			
N3—C1—C2—N1	0.06 (19)	N4—C13—C14—C15	178.33 (15)
C3—C1—C2—N1	178.62 (16)	C13—C14—C15—C10	0.3 (3)
C2—C1—C3—C4	125.1 (2)	C11—C10—C15—C14	1.0 (2)
N3—C1—C3—C4	−56.5 (2)	N1—C10—C15—C14	−178.83 (15)
C1—C3—C4—C9	108.8 (2)	C1—C2—N1—N2	−0.02 (18)
C1—C3—C4—C5	−70.5 (2)	C1—C2—N1—C10	−178.32 (15)
C9—C4—C5—C6	−1.8 (3)	C11—C10—N1—C2	−14.9 (2)
C3—C4—C5—C6	177.62 (17)	C15—C10—N1—C2	164.92 (16)
C4—C5—C6—C7	0.2 (3)	C11—C10—N1—N2	166.96 (15)
C5—C6—C7—C8	1.5 (3)	C15—C10—N1—N2	−13.2 (2)
C6—C7—C8—C9	−1.6 (3)	C2—N1—N2—N3	−0.03 (18)
C5—C4—C9—C8	1.7 (3)	C10—N1—N2—N3	178.44 (14)
C3—C4—C9—C8	−177.68 (16)	N1—N2—N3—C1	0.07 (19)
C7—C8—C9—C4	−0.1 (3)	C2—C1—N3—N2	−0.1 (2)
C15—C10—C11—C12	−1.7 (3)	C3—C1—N3—N2	−178.76 (16)
N1—C10—C11—C12	178.13 (16)	C12—C13—N4—O2	−179.00 (18)
C10—C11—C12—C13	1.0 (3)	C14—C13—N4—O2	1.6 (3)
C11—C12—C13—C14	0.3 (3)	C12—C13—N4—O1	0.4 (3)
C11—C12—C13—N4	−179.03 (16)	C14—C13—N4—O1	−178.99 (19)
C12—C13—C14—C15	−1.0 (3)		

### Hydrogen-bond geometry (Å, º)

Cg1–Cg3 are the centroids of the N1–N3/C1/C2, C4–C9 and C10-C15 rings, respectively.

**Table:**

*D*—H···*A*	*D*—H	H···*A*	*D*···*A*	*D*—H···*A*
C3—H3*B*···O2^i^	0.97	2.58	3.452 (3)	150
C3—H3*A*···*Cg*2^ii^	0.97	2.96	3.857 (2)	154
C8—H8···*Cg*1^iii^	0.93	2.86	3.665 (3)	146
N4—O1···*Cg*3^iv^	1.21 (1)	3.67 (1)	4.1254 (19)	103 (1)

Symmetry codes: (i) −*x*+1, −*y*, −*z*+2; (ii) *x*−1, *y*, *z*; (iii) −*x*+2, *y*−1/2, −*z*+3/2; (iv) −*x*+1, −*y*+1, −*z*+2.
